# A polymorphism in the gene of the endocannabinoid-degrading enzyme FAAH (*FAAH C385A*) is associated with emotional–motivational reactivity

**DOI:** 10.1007/s00213-012-2785-y

**Published:** 2012-07-10

**Authors:** Annette Conzelmann, Andreas Reif, Christian Jacob, Peter Weyers, Klaus-Peter Lesch, Beat Lutz, Paul Pauli

**Affiliations:** 1 Department of Psychology (Biological Psychology, Clinical Psychology, and Psychotherapy), University of Würzburg, Marcusstr 9-11, 97070 Wurzburg, Germany; 2 Department of Psychiatry and Psychotherapy, University of Würzburg, Wurzburg, Germany; 3 Institute of Physiological Chemistry, University Medical Center of the Johannes Gutenberg University Mainz, Mainz, Germany

**Keywords:** FAAH, Genetics, Endocannabinoid, Emotion, Startle reflex

## Abstract

**Rationale:**

The endocannabinoid (eCB) system is implicated in several psychiatric disorders. Investigating emotional–motivational dysfunctions as underlying mechanisms, a study in humans revealed that in the C385A polymorphism of the fatty acid amide hydrolase (FAAH), the degrading enzyme of the eCB anandamide (AEA), *A* carriers, who are characterized by increased signaling of AEA as compared to *C*/*C* carriers, exhibited reduced brain reactivity towards unpleasant faces and enhanced reactivity towards reward. However, the association of eCB system with emotional–motivational reactivity is complex and bidirectional due to upcoming compensatory processes.

**Objectives:**

Therefore, we further investigated the relationship of the FAAH polymorphism and emotional–motivational reactivity in humans.

**Methods:**

We assessed the affect-modulated startle, and ratings of valence and arousal in response to higher arousing pleasant, neutral, and unpleasant pictures in 67 *FAAH C385A* *C*/*C* carriers and 45 *A* carriers.

**Results:**

Contrarily to the previous functional MRI study, *A* carriers compared to *C*/*C* carriers exhibited an increased startle potentiation and therefore emotional responsiveness towards unpleasant picture stimuli and reduced startle inhibition indicating reduced emotional reactivity in response to pleasant pictures, while both groups did not differ in ratings of arousal and valence.

**Conclusions:**

Our findings emphasize the bidirectionality and thorough examination of the eCB system’s impact on emotional reactivity as a central endophenotype underlying various psychiatric disorders.

**Electronic supplementary material:**

The online version of this article (doi:10.1007/s00213-012-2785-y) contains supplementary material, which is available to authorized users.

## Introduction

The endocannabinoid (eCB) system is associated with psychiatric disorders which are marked by emotional–motivational dysfunction, e.g., anxiety, addiction, eating disorders, and depression (Davis 2008; Monteleone et al. 2009; Parolaro et al. 2010; Sipe et al. 2002), and it constitutes an important research topic in relation to psychiatric disorders (Lutz 2009).

The impact of the eCB system on emotional behavior is very likely due to the central role of the eCB anandamide (AEA). Like the phytocannabinoid Δ^9^-tetrahydrocannabinol, AEA activates the cannabinoid type 1 (CB1) receptor as the most important binding sites in brain regions involved in regulation of emotions (Mackie 2005). AEA is degraded by the enzyme fatty acid amide hydrolase (FAAH; Cravatt et al. 1996), which also is a promising target for pharmacological interventions in the treatment of the psychiatric disorders mentioned above (Justinova et al. 2008; Monteleone et al. 2009; Schacht et al. 2009; Sipe et al. 2002). Accordingly, FAAH should play a key role in AEA-mediated emotional effects.

One recent seminal study (Hariri et al. 2009) tested the assumed association of FAAH and emotional–motivational reactivity in humans by investigating the influence of the genetic *FAAH C385A* polymorphism on activity in the brain’s emotion centers. The *A* allele variant of this polymorphism is associated with reduced levels of FAAH protein as compared to the *C*/*C* variant, as the *A* variant displays normal catalytic properties, but an enhanced sensitivity to proteolytic degradation (Chiang et al. 2004; Sipe et al. 2002), presumably leading to a shorter half-life time, and thus to increased AEA signaling. The brain activities of *A* and *C*/*C* carriers registered during a face allocation and a gambling task were compared. In the face allocation task, participants saw two faces (angry and fearful) and had to indicate the one resembling a third target face; a comparable task with shapes served as a control condition. In the gambling task, participants had to indicate whether the value of a card was higher or lower than 5 followed by positive or negative feedback and the requirement to press a button after the positive feedback. They had the possibility to win money. This was compared to a control task, where alternating button presses during the presentation of an “x” which was followed by an asterisk and a yellow circle were required. Results indicated for *FAAH C385A A* compared to *C*/*C* carriers an enhanced reactivity of the ventral striatum in the gambling task and a reduced reactivity of the amygdala in the face allocation task (Hariri et al. 2009). Results seem to indicate that FAAH *A* carriers are characterized by a reduced reactivity towards threat and an increased reactivity towards reward.

These results are in line with reports that disruption of FAAH activity may have anxiolytic and antidepressant effects mediated by CB1 receptor stimulation (Bortolato et al. 2007; Kathuria et al. 2003; Moreira et al. 2008). Possibly, anxiolytic effects by *FAAH C385A* are mediated by arousal, which is increased in *C*/*C* carriers (Dlugos et al. 2010). In addition, stimulation of the CB1 receptor by eCBs, e.g., by decreasing FAAH enzymatic activity, has reinforcing effects and can induce happiness, food intake, and drug seeking by increasing dopamine activity in the nucleus accumbens (Basavarajappa et al. 2006; Mahler et al. 2007; Pagotto et al. 2006; Solinas et al. 2007; Tanda and Goldberg 2003). Furthermore, using functional MRI (fMRI) in humans, cannabinoids were found to reduce amygdala reactivity towards threat (Phan et al. 2008).

However, even though the study of Hariri et al. (2009) has importantly advanced our knowledge about FAAH-modulated brain responses triggered by emotional–motivational stimuli, further studies are needed for several reasons. First, Hariri et al. (2009) examined FAAH-modulated brain reactivity in response to emotional salient stimuli which were part of specific tasks; thus, FAAH effects on the “pure” processing of emotional stimuli independent of other cognitive processes need to be examined. Second, Hariri et al. (2009) examined responses to specific low-arousing stimuli, i.e., faces expressing anger and threat or stimuli signaling positive and negative feedback, although emotional stimuli may vary on a valence dimension from pleasant to unpleasant and also with respect to arousal values. Third, the eCB system’s involvement in emotional processing is very complex and also bidirectional (Moreira and Lutz 2008), which requires further studies to this theme using various stimuli and study conditions. Finally, it seems desirable to complement the work of Hariri et al. (2009) on *FAAH C385A* polymorphism and emotional–motivational reactivity by further experiments using behavioral measures of emotional reactivity; fMRI measures involve methodological concerns including baseline problems (Canli and Lesch 2007) and a lack of validated animal models. Further studies warranted aim at using measures of emotional reactivity which allow translational research necessary to unravel the neurochemical pathway underlying the eCB effects on emotions, and in further perspectives to address clinical pharmacological questions of treatment and prevention.

A behavioral measure directly indicating emotional reactivity in animal and human is the affect-modulated startle reflex. In humans, this reflex is assessed by the eye blink via EMG at the M. orbicularis oculi elicited by a load abrupt noise (Blumenthal et al. 2005). It is well known that the defensive startle reflex is potentiated during the presentation of unpleasant stimuli because of the engagement of the defensive system. On the other hand, the startle reflex is attenuated during pleasant stimuli because of the mismatch between the defensive reflex and the engaged appetitive system (Lang 1995). Addiction and anxiety disorders were observed to be related to specific abnormalities in startle reflex modulation (Grillon and Baas 2003).

Accordingly, the aim of this study was to further investigate the influence of the *FAAH C385A* polymorphism on emotional–motivational reactivity using a translational behavioral measure, the affect-modulated startle reflex, which was assessed during the presentation of pleasant, neutral, and unpleasant International Affective Picture System (IAPS) pictures (Lang et al. 2005). Based on the study by Hariri et al. (2009), *A* allele carriers were expected to show a reduced reactivity towards unpleasant stimuli and an increased reactivity towards pleasant stimuli as compared to *C*/*C* carriers.

## Materials and methods

### Participants

Data of 112 adult participants from the general population were analyzed (67 *FAAH C385A* *C*/*C* carriers and 45 *A* carriers). Originally, the sample consisted of 119 participants. However, seven participants had to be excluded as startle nonresponders (seven *C*/*C* carriers and two *A* carriers; deviation of the number of startle zero reactions of more than 2.5 standard deviations of the sample’s mean). A further description of the sample can be found in the “Results” section.

All participants were recruited via newspaper advertisement. After a telephone screening ensuring the absence of current or lifetime psychiatric disorders or severe somatic disorders, participants completed a diagnostic interview for psychiatric disorders [Structured Clinical Interview for DSM-IV Axis I Disorders (SCID-I) and SCID II (First 1996; First et al. 1997)]. Only participants free of any lifetime or present psychiatric diagnoses and free from current drug or alcohol consumption were recruited. Further exclusion criteria were the age under 18 and over 60 years, IQ level below 80 [MWT-B (Lehrl 1989)], severe somatic disorders, hearing problems, and psychotropic medication. Written informed consent, according to the guidelines of the ethical committee in Würzburg and the ethical standards laid down in the 1964 Declaration of Helsinki, was provided by all participants.

### Genotyping

DNA has been extracted from peripheral blood by a routine desalting method. rs324420 has been determined by standard polymerase chain reaction. The following primers were used to amplify a region of 333 bp: F:5′-TGTTGCTGGTTACCCCTCTC-3′ and R:3′-AATGACCCAAGATGCAGAGC-5′. The PCR reaction mix (25 μl) consisted of 10× Goldstar mix (2.5 μl), 15 mM MgCl_2_ (1 μl), 2.5 mM of each nucleotide (1 μl), 1 μl of each primer, 0.2 μl Taq Polymerase, 18.3 μl of ddH_2_O, and 1 μl DNA template. Cycling conditions were 95 °C denaturation at 5 min, then 35 cycles of 45 s at 95 °C/45 s at 58.8 °C/45 s at 72 °C. The final step was 5 min at 72 °C. PCR product was digested at 37 °C with STYI (New England Biolabs, Beverly, MA, USA) which cuts the amplicon in the presence of the ancestral C allele in fragments of 200 and 133 bp. Fragments were visualized on an agarose gel stained with ethidium bromide.

### Procedure

The experimental procedures were controlled by Experimental Run Time System (version 3.32, BeriSoft Cooperation, Frankfurt, Germany). The study began with a familiarization to the experimental protocol by presenting two pleasant, two neutral, and two unpleasant pictures from the IAPS (Lang et al. 2005); all picture numbers can be found in Electronic supplementary material 1) with startle tones (50 ms of 95-dB white noise with an instantaneous rise time presented binaurally with Beyerdynamic DT 331 headphones, Heilbronn, Germany) presented during one picture of each valence category. During the experiment proper, 18 pleasant, 18 neutral, and 18 unpleasant IAPS pictures were presented together with startle tones; 18 additional pictures, six of each valence category, were used as fillers. Pictures were shown for 8 s with an inter-trial interval (ITI) of 16.5–25.5 s. The startle tones were administered 2.5, 4.0, or 5.5 s after picture onset. Eighteen additional startle tones during the ITIs were delivered to measure baseline startle responses and startle habituation. Startle stimuli were displayed on the background of a constant 60-dB white background noise (white noise generator Lafayette Instruments Co., Lafayette, IN). To ensure comparable valence and arousal levels, some pictures were different for men and women (81 % were exactly the same or were matched for content). For females and males, three pseudorandomized picture orders with not more than two consecutive pictures of the same valence or the same startle onset time were generated. After the startle assessment, pictures were presented again in a free-viewing condition, and participants rated the pictures’ valence and arousal using nine-point Self-Assessment Manikin scales (Lang 1980).

### Physiological recordings

EMG was measured from the left M. orbicularis occuli with a Vitaport II system (Becker Meditec, Karlsruhe, Germany; 512 Hz with online high- and low-pass filters of 0.015 s and 2,200 Hz). The signals were rectified, integrated, stored at 256 Hz, and later smoothed offline (using a time constant of 100 ms) and analyzed by one experienced expert blind to genetic condition using Matlab (The MathWorks, München, Germany). Startle magnitude was the difference between the peaks in a time window of 19.5–151.5 ms after the onset of the startle tone minus the baseline as mean EMG over 19.5 s before the startle stimulus. Trials with baseline shifts from 19.5 ms before to 15.6 ms after the startle probe were excluded from the analysis. Trials with no detectable peak were scored as zero.

### Data analyses

Sociodemographic data were evaluated with one-way ANOVAs with genotype (*FAAH C385A* *C*/*C* carrier and *FAAH C385A* *A* carrier) and sex as between-subjects factors, or with *χ* ^2^ tests. For affect-modulated startle response as well as valence and arousal ratings, means were calculated for each valence condition (pleasant, neutral, and unpleasant; for startle data, means were built over all startle onsets per valence condition^1^). These means were then entered into repeated-measures ANOVAs with genotype and sex as between-subjects factors and picture valence (pleasant, neutral, and unpleasant) as a within-subjects factor. Startle habituation was analyzed with a repeated-measures ANOVA with genotype and sex as between-subjects factors and time of measurement (T1 to T6; each as mean over three consecutive ITI startle stimuli) as a within-subjects factor. Sex was included in all analyses to control for genotype x sex effects. However, sex never had an impact, and sex effects are therefore not reported. Greenhouse–Geisser corrections were used if appropriate. Post hoc tests reflect Bonferroni–Holm corrected *p* values.

## Results

### Sample characteristics

*FAAH C385A* allele frequencies were 0.78 for C and 0.22 for A. The genotype distribution (*C*/*C* = 67, *C*/*A* = 40, and *A*/*A* = 5) was in accordance with the Hardy–Weinberg equilibrium (*χ* ^2^ = 0.1, n.s.). There were no differences between the *FAAH C385A* *A* variant and *C*/*C* variant in sex distribution (*χ* ^2^(1) = 0.3, *p* = 0.264), age, IQ, trait anxiety, impulsivity, empathy, or venturesomeness (all *F*s < 2.1, *p*s > 0.145; see Table 1).

**Table 1:** Participant characteristics

	*FAAH C385A* A carriers	*FAAH C385A C* / *C* carriers
	Mean ± SD or *n*	Mean ± SD or *n*
Sex (*n* women/*n* men)	27/18	33/34
Age	34.4 ± 9.5	34.2 ± 10.2
IQ^a^	116.8 ± 13.0	117.9 ± 14.2
STAI^b^ Trait Anxiety	33.0 ± 6.7	33.3 ± 8.9
I7^c^ Impulsivity	5.1 ± 2.8	6.0 ± 3.1
I7^c^ Empathy	13.9 ± 3.4	13.9 ± 3.0
I7^c^ Venturesomeness	8.2 ± 3.3	8.8 ± 3.3
Valence/arousal rating^d^		
Pleasant pictures	7.2 ± 0.7/5.0 ± 1.1	7.0 ± 0.7/5.0 ± 1.0
Neutral pictures	5.4 ± 0.8/3.8 ± 1.0	5.3 ± 0.7/3.6 ± 1.1
Unpleasant pictures	2.6 ± 0.8/6.6 ± 1.0	2.6 ± 0.7/6.6 ± 0.9

^a^Intelligence quotient assessed with the MWT-B (Lang 1980)

^b^Stait Trait Anxiety Inventory (Spielberger et al. 1970)

^c^I7 questionnaire (Eysenck et al. 1985)

^d^Scales: valence, 1 = highly unpleasant, 9 = highly pleasant; arousal, 1 = calm, 9 = excited

### Picture ratings

Mean picture ratings stratified for the gene variants can be derived from Table 1. ANOVAs indicated significant effects for the valence [*F*(2, 216) = 1,428.0, *p* < 0.001] and the arousal [*F*(2, 216) = 404.0, *p* < 0.001] ratings. The valence rating increased from unpleasant to neutral to pleasant pictures (all *t*s(111) > 22.6, *p*s < 0.001; mean ± SD, pleasant, 7.1 ± 0.7; neutral, 5.4 ± 0.8; unpleasant, 2.6 ± 0.7). The arousal was rated higher for emotional compared to neutral, and for unpleasant compared to pleasant pictures (all *t*s(111) > 12.4, *p*s < 0.001; mean ± SD, pleasant, 5.0 ± 1.1; neutral, 3.7 ± 1.0; unpleasant, 6.6 ± 0.9). There were no effects of genotype or genotype × valence [all *F*s < 1.6, *p*s > 0.198].

### Startle modulation

**Fig. 1:**
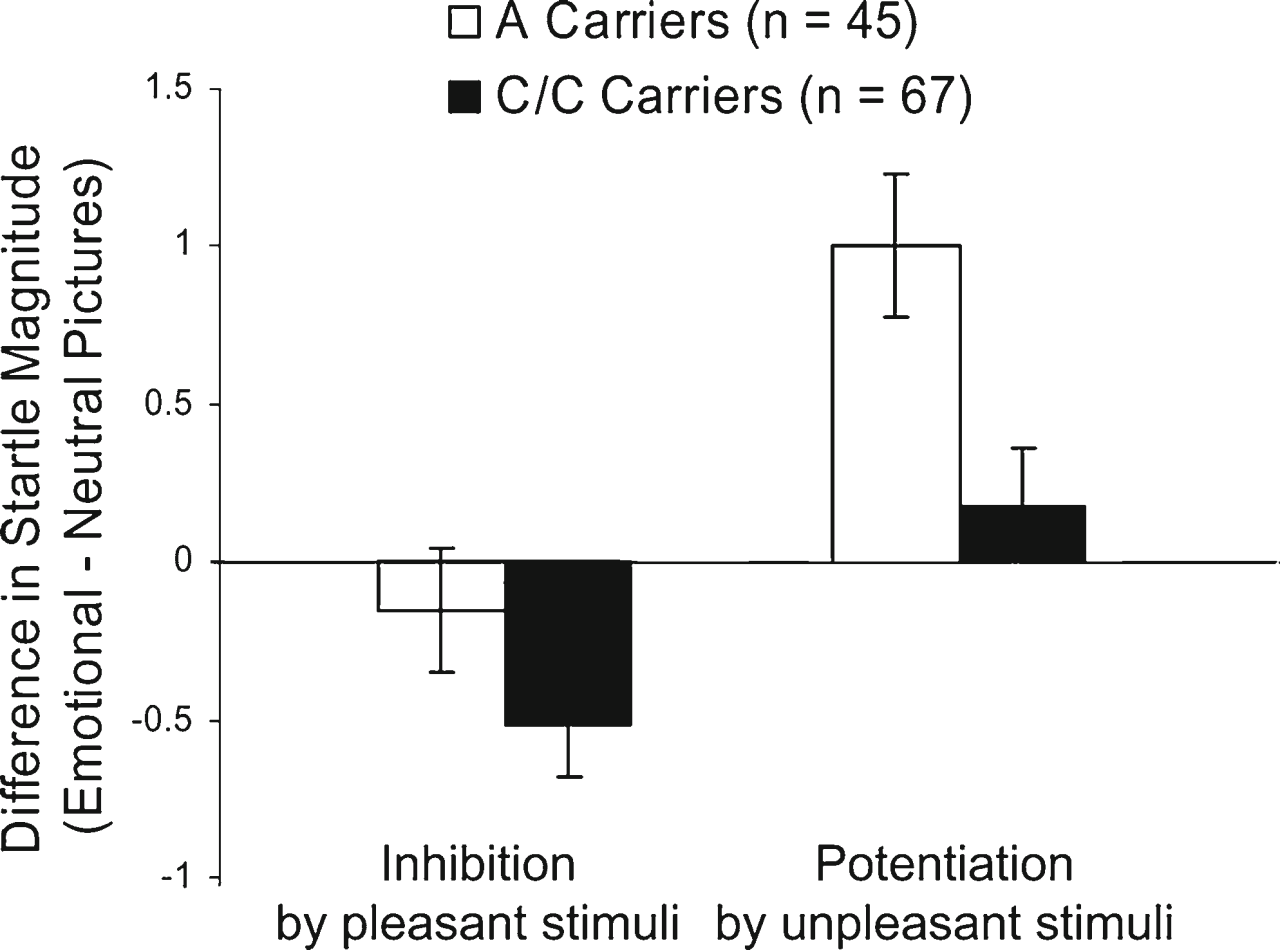
Means (±SE) for *FAAH C385A* A carriers (*n* = 45) and CC carriers (*n* = 67) of affect-modulated startle inhibition during pleasant and startle potentiation during unpleasant pictures (difference scores emotional–neutral pictures)

The ANOVA for the affect-modulated startle data revealed a main effect of valence [*F*(2, 216) = 22.2, *p* < 0.001], which was due to increasing startle responses from pleasant to neutral to unpleasant pictures (all *t*s(111) > 3.0, *p*s < 0.004). There was no main effect for genotype [*F*(1, 108) = 0.1, *p* = 0.772], but a genotype × valence interaction [*F*(2, 216) = 3.9, *p* = 0.024] (Fig. 1). The genotype × valence interaction was further analyzed by comparing groups regarding startle inhibition by pleasant pictures and startle potentiation by unpleasant pictures (difference emotional minus neutral stimuli). This ANOVA revealed a significant genotype main effect [*F*(1, 110) = 6.9, *p* = 0.010], indicating that *A* carriers as compared to *C*/*C* exhibited enhanced startle potentiation for unpleasant pictures and reduced startle inhibition for pleasant pictures; the genotype × valence interaction was not significant [*F*(1, 110) = 2.2, *p* = 0.145]. *T* tests against zero demonstrated that startle potentiation was significant for *A* [*t*(44) = 4.2, *p* < 0.001] but not *C*/*C* carriers [*t*(61) = 1.1, *p* = 0.568], while startle inhibition was significant for *C*/*C* [*t*(61) = −3.0, *p* = 0.012] but not *A* carriers [*t*(44) = −0.8, *p* = 0.434].^2^

The ANOVA for the ITI startle baseline and its habituation revealed a significant main effect of time [*F*(5, 540) = 53.4, *p* < 0.001], indicating that the startle responses declined during the experiment (all *t*s > 3.0, *p*s < 0.012; except for T3 = T4 and T5 = T6, *t*s < 2.1, *p*s > 0.084; mean ± SD, T1 = 10.2 ± 7.5, T2 = 8.5 ± 7.3, T3 = 6.6 ± 5.9, T4 = 6.4 ± 6.0, T5 = 5.4 ± 5.2, T6 = 4.8 ± 5.1). Neither the genotype main effect nor the genotype × time interaction was significant [all *F* < 0.5, *p*s > 0.486].

## Discussion

This study, to our knowledge, is the second directly assessing the association of the *FAAH* polymorphism (C385A) with emotional–motivational responses in humans. The preceding study by Hariri et al. (2009) revealed in *FAAH C385A* *A* as compared to *C*/*C* carriers reduced amygdala reactivity during a face allocation task involving fearful and angry faces and an increased ventral striatum reactivity in a gambling task with positive and negative feedback and the possibility to win money. We wished to provide new data demonstrating effects of the *FAAH C385A* polymorphism on the engagement of the defensive and appetitive motivational systems towards stimuli from the IAPS varying on the valence dimension from pleasant over neutral to unpleasant, as reflected in behavioral measure, i.e., startle response potentiation and inhibition, respectively.

While self-reports of valence and arousal were not affected by genotype, we observed in *FAAH C385A* *A* carriers as compared to *C*/*C* carriers both an increased startle potentiation indicating an increased reactivity towards unpleasant pictures, as well as a reduced startle inhibition reflecting a reduced reactivity towards pleasant pictures. These behavioral results suggest enhanced amygdala and reduced ventral striatum activity in *FAAH C385A* *A* compared to *C*/*C* carriers in response to unpleasant or pleasant stimuli, respectively (see Koch 1999). Accordingly, our results stand in contrast to the only other study on this topic (Hariri et al. 2009).

Interestingly, there are studies supporting both directions of how *FAAH C385A* may influence emotional–motivational reactivity. While several studies on anxiolytic and reinforcing effects of FAAH disruption and increased eCB signaling, respectively, are in accordance with the Hariri et al. (2009) findings (e.g., Basavarajappa et al. 2006; Bortolato et al. 2007; Kathuria et al. 2003; Mahler et al. 2007; Moreira et al. 2008; Pagotto et al. 2006; Solinas et al. 2007; Tanda and Goldberg 2003), some other studies observed the opposite association in line with our results (e.g., Hall and Solowij 1998; Kalant 2004; Moreira et al. 2009; Morrison et al. 2009; Trezza and Vanderschuren 2009).

It may be speculated that this bidirectionality is related to the fact that a low activation of the eCB system and AEA signaling have anxiolytic effects via CB1 receptor activation, while high activation increases anxiety and aversion by binding of AEA to transient receptor potential vanilloid 1 channel triggering a downregulation of the eCB 2-arachidonoyl glycerol (Kathuria et al. 2003; Maccarrone et al. 2008; Moreira et al. 2008; Rubino et al. 2007, 2008; Ruehle et al. 2012; Sañudo-Peña et al. 1997; Terzian et al. 2009; Viveros et al. 2005). While *FAAH C385A* *A* carriers should be characterized by increased levels of the eCB AEA as compared to *C*/*C* carriers (Chiang et al. 2004; Sipe et al. 2002), AEA levels should additionally depend on the intensity of the emotional context (Marsicano et al. 2002). Therefore, low-arousing contexts as used in the Hariri et al. (2009) study (angry and fearful faces, Wieser et al. 2011) or reward as part of possibly distracting cognitive–motor tasks) may lead to anxiolytic effects, while high arousing stimuli (pleasant, neutral, and unpleasant images of various contents and without cognitive load) as used in the present study may have anxiogenic effects. However, this speculation must be experimentally tested in future studies using differentially arousing emotional contexts, different assessment methods, and if possible pharmacological receptor blockers.

Interestingly, both the present and the Hariri et al. (2009) study observed that the association of the *FAAH C385A* polymorphism with emotional responses was contrary for unpleasant and pleasant stimuli. This suggests that the differential effects of *FAAH C385A* on responsiveness towards pleasant and unpleasant stimuli are based on connections between the amygdala and the ventral striatum (Yim and Mogenson 1982, 1989).

Of course, our study also has limitations. First, the study is correlative and cannot deliver answers about the nature of the association between *FAAH C385A* and emotional reactivity. Second, comparably to the study of Hariri et al. (2009), the polymorphism did not influence the verbally assessed phenotypes, such as impulsivity, anxiety or valence, and arousal ratings. However, ratings and verbal responses are much more prone to be biased by normative behavior, expectancies, and social requirements, and endophenotypes such as emotion reactivity may be better revealed by biopsychological measures (Castellanos and Tannock 2002). Third, we examined a truly healthy sample on the basis of a thorough screening for any psychopathology. Thus, we purposely decreased the variance of the phenotype, and as a consequence, reduced the power to detect genetic effects. Since Hariri et al. (2009) did not explicitly screen for psychopathology in the past, we have to assume that their sample included some participants with at least lifetime axis I disorders. However, this difference in sample characteristics very unlikely can explain the opposite findings.

The eCB system is involved in various psychiatric disorders, such as mood disorders, addiction, pain, and anxiety disorders (Monteleone et al. 2009; Parolaro et al. 2010; Sipe et al. 2002). FAAH inhibitors are promising pharmacological agents in the treatment of pain and anxiety without drug-seeking side effects (Davis 2008). Accordingly, this study contributes to an area of substantial interest for the development of psychological and psychopharmacological treatments and asks for further research to clarify the bidirectionality of the eCB system’s influence on emotion processing.

In summary, we believe to have assessed emotional reactivity with an objective, reliable, and valid experimental protocol. Startle responses were scored by one experienced expert blind to the genetic conditions, and picture ratings as well as general startle effects, such as the observed main valence and habituation effects, are in accordance with a multitude of previous studies. Therefore, our results justify a new starting point for further research on the association between the FAAH enzyme and the eCB neurotransmitter system and emotional–motivational reactivity. We are far away with our study from explaining the exact mechanism of this association. However, we hope that we could indicate that a one-sided view of this association is not sufficient, and further research is needed.

## Electronic supplementary material

Below is the link to the electronic supplementary material.

ESM 1 DOC 30 kb

## Footnotes

1 There was no influence of startle onset on valence or genotype effects. This was verified by an additional ANOVA including startle onset as additional within-subjects factor together with valence, sex, and genotype. The valence and genotype x valence effects remained (*F*s > 3.6, *p*s < 0.028), while all other effects including startle onset and valence or genotype were not significant (*F*s < 2.0, *p*s > 0.104).

2 To take measures of impulsiveness and trait anxiety into account, both variables were entered as covariates into the overall ANOVA for affect-modulated startle response. The genotype × valence interaction remained significant [*F*(2, 212) = 3.9, *p* = 0.025]. In addition, we found no correlations between startle potentiation and startle inhibition and measures of anxiety and impulsiveness (all *p* > 0.085).
