# Supplementary material for: A polymorphism in the gene of the endocannabinoid-degrading enzyme FAAH (FAAH C385A) is associated with emotional–motivational reactivity
Source: Psychopharmacology (Berl). 2012 Jul 10;224(4):573–9. doi: 10.1007/s00213-012-2785-y (PMC3505526; doi:10.1007/s00213-012-2785-y)
Supplement: Supplementary file 1 — DOC 30 kb [file 213_2012_2785_MOESM1_ESM.doc]

**Appendix**

**IAPS picture numbers**

72 pictures were taken from the International Affective Pictures System (IAPS): Women: 7820, 6160, 2120, 5460, 9920, 3170, 7010, 7200, 1811, 7190, 4660, 2057, 7020, 3150, 7050, 2205, 9520, 7175, 2221, 4680, 8370, 7000, 7205, 8030, 5830, 9700, 4640, 4641, 1270, 5533, 6550, 7270, 4510, 7004, 7237, 6312, 5621, 6530, 6241, 9250, 5629, 7950, 9160, 3062, 2750, 8490, 1090, 2691, 4613, 8180, 1590, 2720, 2200, 7185; Filler Pictures: 9910, 7182, 7160, 5480, 3080, 7283, 9620, 7002, 5660, 1540, 4572, 8034, 7031, 9008, 6360, 9440, 2480, 8496. Men: 7820, 6160, 2120, 5460, 9920, 3170, 7010, 7200, 1811, 7190, 4660, 2057, 7020, 3150, 7050, 2205, 9520, 7175, 2221, 4680, 8370, 7000, 7205, 8030, 5830, 9700, 4650, 4607, 1274, 5534, 3530, 7330, 4290, 7080, 7187, 6370, 8080, 3010, 6230, 3140, 5260, 7110, 9410, 3000, 4180, 2053, 6150, 3103, 7180, 5700, 7550, 8510, 7130, 9040; Filler Pictures: 9910, 7182, 7160, 5480, 3080, 7283, 9620, 7002, 5982, 1460, 4420, 8380, 7090, 9007, 9433, 9810, 4310, 5740. In the pleasant picture category, there were pretty animals, babies, erotic scenes, landscapes, sports and leisure activities, and food. For the neutral category there were neutral faces, mushrooms, and household appliances. Unpleasant stimuli were pictures of threatening animals and people, angry faces, corpses, mutilation, weapons, pollution, and accidents.
